# Chest physiotherapy in preterm infants with lung diseases

**DOI:** 10.1186/1824-7288-36-65

**Published:** 2010-09-26

**Authors:** Carmen Giannantonio, Patrizia Papacci, Roberta Ciarniello, Mikael Ghennet Tesfagabir, Velia Purcaro, Francesco Cota, Carla Maria Semeraro, Costantino Romagnoli

**Affiliations:** 1 Department of Paediatrics, Division of Neonatology, "Sacro Cuore" Catholic University, Largo Francesco Vito 1, Rome, Italy; 2 Department of Physiatrics, Service of Physical Medicine and Rehabilitation, " Sacro Cuore" Catholic University, Largo Francesco Vito 1, Rome, Italy

## Abstract

**Background:**

In neonatology the role of chest physiotherapy is still uncertain because of the controversial outcomes.

**Methods:**

The aim of this study was to test the applicability in preterm infants of 'reflex rolling', from the Vojta method, in preterm neonates with lung pathology, with particular attention to the effects on blood gases and oxygen saturation, on the spontaneous breathing, on the onset of stress or pain. The study included 34 preterm newborns with mean gestational age of 30.5 (1.6) weeks - mean (DS) - and birth weight of 1430 (423) g - mean (DS) -, who suffered from hyaline membrane disease, under treatment with nasal CPAP (continuous positive airways pressure), or from pneumonia, under treatment with oxygen-therapy. The neonates underwent phase 1 of 'reflex rolling' according to Vojta method three times daily. Respiratory rate, SatO_2_, transcutaneous PtcCO_2 _e PtcO_2 _were monitored; in order to evaluate the onset of stress or pain following the stimulations, the NIPS score and the PIPP score were recorded; cerebral ultrasound scans were performed on postnatal days 1-3-5-7, and then weekly.

**Results:**

In this population the first phase of Vojta's 'reflex rolling' caused an increase of PtcO_2 _and SatO_2 _values. No negative effects on PtcCO_2 _and respiratory rate were observed, NIPS and PIPP stress scores remained unmodified during the treatment; in no patient the intraventricular haemorrhage worsened in time and none of the infants developed periventricular leucomalacia.

**Conclusions:**

Our experience, using the Vojta method, allows to affirm that this method is safe for preterm neonates, but further investigations are necessary to confirm its positive effects and to evaluate long-term respiratory outcomes.

## Background

Chest physiotherapy (CPT) has been used to clear secretions, to reduce post-extubation atelectasis, to reduce the use of reintubation, and also to help lung ventilation in newborns with respiratory problems [1-3]. However, concerns about the safety of some forms of chest physiotherapy have been raised, especially for very low birth weight infants (VLBW), due to the risk of brain damage related to some CPT techniques [4].

The forms of CPT more commonly used during the neonatal period are active chest physiotherapy (tapping or vibration delivered on the chest) and non-active techniques (e.g. positioning and suction alone), but caution is required also when interpreting the possible positive effects of these chest physiotherapy treatments [5-8].

With regard to the different CPT techniques and the controversial outcomes they yield, we decided to test the applicability in preterm infants of 'reflex rolling', from the Vojta method.

The Vojta method is a physical therapy, initially developed in the 1960 s for the treatment of children with or at risk of cerebral palsy. It is a program that employs isometric strengthening techniques through tactile stimulation, to encourage the development of normal movement patterns and therefore to improve respiration. The aim of this study was to evaluate the 'safety' of Vojta reflex stimulations in preterm neonates with lung diseases, investigating particularly the effects on blood gases and on oxygen saturation, the effects on spontaneous breathing and the presence of stress/pain.

## Methods

The Ethical Committee of the Paediatrics Department approved the study protocol, and parents gave their informed consent.

### Subjects

The study included preterm newborns with gestational age ranging from 28 to 34 weeks, admitted to the Neonatal Intensive Care Unit of "A.Gemelli" Hospital (Sacro Cuore Catholic University, Rome), from 1 January 2008, to 30 September 2008, who suffered from hyaline membrane disease, under treatment with NCPAP (nasal continuous positive airways pressure), or from pneumonia, who received oxygen-therapy.

Newborns with congenital malformations, asphyxia at time of birth, under treatment with neurotropic drugs, or with intraventricular haemorrhage > 2^nd ^grade according to Papile's classification, were excluded from the study [9].

All newborns breathed spontaneously, and were treated with:

1. Continuous positive pressure ventilation delivered by nasal cannula (maximum CPAP = 6 cm H_2_O and maximum FiO_2 _= 0.40)

2. Oxygen-therapy with maximum FiO_2 _= 0.40

After birth, the newborns received citrate caffeine as prophylaxis of apnoea of prematurity. Antibiotic therapy was administered when diagnosis of pneumonia (clinical and radiological) was made.

#### The 'reflex rolling' according to Vojta

The neonates underwent phase 1 of ***reflex rolling ***according to Vojta. This manoeuvre does not require the newborn to be moved, but only a slight rotation of the head towards the side from which the stimulus is delivered. The starting position for performing the first phase of reflex rolling is the asymmetric supine position, with the limbs freely lying on the resting surface.

A digitopressure was exerted on the chest area, where the mammillary line crosses the insertion of the diaphragm, either at the level of the 6^th ^rib, or between the 5^th ^and the 6^th^, or between the 6^th ^and the 7^th^.

Each treatment consisted in delivering four stimuli, two to the left half of the chest (stimulations I and II) and two to the right half of the chest (stimulations III and IV). Each stimulus consisted of a slight pressure, progressively oriented in dorsal, medial and cranial directions, diagonally to the spine. The treatment was repeated three times a day, at time intervals of 0, 2 and 4 hours.

### Monitoring and controls

The tests performed were the following:

a. Respiratory rate (RR) and SatO_2 _were monitored by Hewlett-Packard HP monitor (Hewlett Packard M1205AOmni Care model, Andover, Germany);

b. Transcutaneous monitoring of PtcCO_2 _e PtcO_2 _by TINA (Radiometer Medical, Copenhagen, Denmark).

The tests were performed before the reflex stimulation, at the end of stimulation II, at the end of stimulation IV and at 5, 15, 25 minutes after the whole series of stimulations, during each of the three daily treatments.

c. Cerebral ultrasound scan on postnatal days 1-3-5-7, and weekly, using a color Doppler unit. (Hewlett-Packard Doppler 'IMAGE POINT')

#### Monitoring of stress or pain

In order to evaluate the onset of stress or pain following the stimulations, two pain and stress evaluation scales were adopted: the NIPS score (Neonatal Infant Pain Scale) and the PIPP (Premature Infant Pain Profile) Score [10,11].

The NIPS scores were recorded before the reflex stimulation, at the end of the second stimulation, at the end of the fourth stimulation and at 5, 15, 25 minutes after the stimulation, during all three sessions. The PIPP scores were recorded only once in each session during stimulation.

### Statistical Analysis

Continuous data are presented as mean ± standard deviation. To compare each parameter at each time point, Repeated Measures One-way ANOVA with Bonferroni's Multiple Comparison Test was performed using GraphPad Prism version 4.00 for Windows (GraphPad Software, San Diego California USA, http://www.graphpad.com). A score of p < 0.05 was considered significant.

## Results

Over the time of study, 60 neonates showed criteria of eligibility; 7 newborns with severe congenital malformation and 19 newborns who required mechanical ventilation were excluded. Therefore, the neonates included in the study were 34, 19 female and 15 male.

The gestational age of the newborns included in the study was 30.5 (1.6) weeks -mean (DS) - and the neonatal weight was 1430 (423) g - mean (DS) -.

We studied the effects of the application of 'reflex rolling' during the first week of life and after the first week of life. Therefore, two groups of newborns were observed (Table 1):

**Table 1:** Clinical Characteristics of the two study groups

	Group 1	Group 2
**Number of patients**	21	13

**Gestational Age **(weeks) mean ± SD	30.3 ± 1.0	30.8 ± 2.3

**Birth Weight **(g) mean ± SD *(range)*	1340 ± 180 *(1010 - 1640)*	1575 ± 635 *(565 - 2480)*

**Sex ratio **(male/female)	1.1	0.44

**Postnatal Age **(days) mean ± SD *(range)*	6 ± 1 *(2 -7)*	10.6 ± 3.5 *(8 - 20)*

**IVH <3 grade **(n°)	2	4

**CLD **n°(%)	3 (14,3)	3 (23,1)

GROUP 1: 21 newborns with hyaline membrane disease during the first week of life. All newborns were treated with NCPAP.

GROUP 2: 13 newborns with a respiratory disease persisting after the first week of life, who were spontaneously breathing and on oxygen-therapy.

In any case, the IVH (intraventricular haemorrhage) diagnosis was made during the infant's first day of life; in no patient the intraventricular haemorrhage worsened in time. None of the infants we observed developed periventricular leucomalacia.

### Monitoring of blood gases and respiratory rate

In group 1, PtcO_2 _was found to be significantly different across the time points (p < 0.0001); post-hoc analysis according to Bonferroni showed a significant difference (p < 0.05) among the mean values of pO_2_: before the stimulation and at 5', 15' e 25'; at the end of stimulation II and at 5', 15' e 25'; at the end of stimulation IV and at 15' and 25'. In a similar way, SatO_2 _was found to be significantly different across the time points (p < 0.001); post-hoc analysis according to Bonferroni showed a significant difference (p < 0.05) among the mean values of SatO_2_: before the stimulation and at 5'; at the end of stimulation II and at 15' and 25'. No statistically significant difference was found for ptcCO_2. _No increase in RR was recorded; therefore, it is possible that the improved oxygenation induced by this method may be due to an increased tidal volume. (Table 2).

**Table 2:** Blood gases, SatO_2 _and respiratory rate (RR) in group 1

	*pre stim*.	*end II stim*.	*end IV stim*	*5 min*	*15 min*	*25 min*
**PtcO_2 _**(mmHg) Mean (± SD)	61.7 (12.9)	61.3 (12.9)	63.2 (12.7)	68.6 (13.7)	70.9 (13.9)	73.1 (13.6)

**SatO_2 _**(%) Mean (± SD)	92.2 (3.9)	92.0 (2.8)	92.5 (3.8)	93.6 (3.3)	94.3 (3.1)	94.0 (3.2)

**PtcCO_2_** (mmHg) Mean (± SD)	42.1 (6.4)	42.2 (7.2)	42.6 (7.7)	43.0 (6.7)	43.6 (6.4)	44.2 (6.9)

**RR **(b/min) Mean (± SD)	42 (10)	48 (13)	47 (15)	45 (10)	45 (11)	46 (12)

In group 2, PtcO_2 _was found to be significantly different across the time points (p < 0.01); post-hoc analysis according to Bonferroni showed a significant difference (p < 0.05) among the mean values of pO_2_: before the stimulation and at 25'; at the end of stimulation II and at 25'; at the end of stimulation IV and at 25'. SatO_2 _was found to be significantly different across the time points (p < 0.05); but Bonferroni's multiple comparison test did not show any significant difference among individual evaluations. No statistically significant difference was found for ptcCO_2_, nor for RR (Table 3).

**Table 3:** Blood gases, SatO_2 _and respiratory rate (RR) in group 2

	*pre stim*.	*end II stim*.	*end IV stim*	*5 min*	*15 min*	*25 min*
**PtcO_2 _**(mmHg) Mean (± SD)	65.8 (12.9)	63.9 (9.6)	64.1 (11.6)	66.8 (10.8)	69.6 (14.5)	73.6 (15.0)

**SatO_2 _**(%) Mean (± SD)	94.7 (3.0)	95.6 (3.2)	95.7 (4.0)	96.8 (2.8)	97.4 (2.6)	97.5 (2.9)

**PtcCO_2_** (mmHg) Mean (± SD)	45.9 (5.9)	45.8 (6.2)	45.5 (5.2)	44.9 (4.9)	44.8 (4.1)	44.9 (4.0)

**RR **(b/min) Mean (± SD)	46 (15)	41 (11)	42 (16)	41 (12)	45 (18)	47 (13)

### Monitoring of stress/pain

The NIPS scores for the two study groups did not show pain nor stress signs during stimulation, in any of the daily settings. Moreover, the PIPP scores showed no stress. The values found in group l were: 6.1 (1.9) -mean (SD) - with the first recording; 6.0 (2.0) with the second recording; 6.2 (1.5) with the third recording and in group 2 they were 6.3 (1.6) with the first recording; 6.2 (1.7) with the second recording and 6.4 (1.8) with the third recording.

## Discussion

In neonatology, respiratory physiotherapy is still controversial [5].

In treating neonates, the most renowned methods are mechanical techniques such as percussion and vibrations, which represent the so-called active respiratory physiotherapy (ARP), postural drainage, tracheal aspiration, elicitation of the cough reflex, respiratory modifications induced by means of posture.

The specialized literature concerning neonates mainly sets its focus on active respiratory physiotherapy and on the specific aspect of preventing post-extubation atelectasis.

With regard to the prevention of post-extubation atelectasis, the strong doubts concerning the actual utility of active respiratory physiotherapy seem to be confirmed; in fact, there is no evidence of a clear benefit in terms of a decreased rate of post-extubation lobar collapse [5-7,12,13]. Besides the scarce benefits, some authors have suggested that ARP might entail the risk of a neurologic damage, especially in neonates whose weight is <1500 grams, a risk which other studies did not confirm [4,8,13-18].

It is not surprising; however, that active respiratory physiotherapy might prove to be an invasive technique for preterm newborns. In fact, it has been stressed that ventilated patients may show irritability, an increased consumption of oxygen, an increased heart rate and arterial blood pressure, a higher rate of gastro-oesophageal reflux and, what represents a high risk for preterm newborns, an increased intracranial pressure [19,20].

In any case, active respiratory physiotherapy and particularly vibrations and percussion are unsuitable for VLBW neonates, due to their physical features; in fact, in these patients the anatomical and physiological features of the ribcage subdue and cancel the effect of the aforesaid techniques, which nevertheless maintain their effectiveness in childhood and in adult age.

Therefore, non-active respiratory physiotherapy (i.e. postural therapy and postural drainage) seems to be the only respiratory physiotherapic technique available for VLBW neonates.

In 1967 Vàclav Vojta developed and made public the reflex rolling model, defining the chest as a crucial area. By stimulating the chest of a child suffering from athetosis and relapsing episodes of pneumonia and atelectasia unaffected by other active respiratory physiotherapic treatments, Vojta noticed a global reaction, consisting of a rotation of the head with flexion of the lower limbs and rotation of the pelvis, opening of the hands and, what is most important, an increase in depth of costal respiration, with an expansion of the ribcage [21].

The importance of these stimulations, especially if repeated, lies in the fact that the afferences due to induced physiologic muscle activity are imprinted in the central nervous system (CNS) and memorized. The CNS is set in an activated state, and the duration of such activation persists for at least half an hour after the stimulation has ended.

Our study aimed at evaluating the use of 'reflex rolling' in preterm neonates suffering from respiratory diseases. In the neonates we studied, the first phase of Vojta's 'reflex rolling' caused an increase of PtcO_2 _and SatO_2 _values, showing a positive action on oxygenation.

No negative effects on PtcCO_2 _were observed, as these values remained constant over the treatment period, and within the normal range. A further confirmation of this technique's safety came from the negative results of the NIPS e PIPP stress scores, which remained substantially unmodified over the treatment.

The positive results obtained should be followed by a further investigation concerning the efficacy of the 'reflex rolling' technique in preterm newborns, by means of a randomized controlled study. The positive effects may be confirmed by the evaluation of respiratory functionality tests and by long-term respiratory outcomes.

## Conclusion

Although the role of CPT in neonates with respiratory diseases remains debated and needs further evaluation, our experience concerning the use of Vojta's method provides a different perspective and takes again into consideration respiratory physiotherapy as a resource for the treatment of neonatal lung diseases. Studies performed on larger series of patients may be able to definitively confirm the effectiveness of Vojta's method as a suitable treatment for neonates, particularly in order to provide a support for all other therapeutic measures based on the use of drugs and ventilation.

## Competing interests

The authors declare that they have no competing interests.

## Authors' contributions

CG: drafted the manuscript, PP: designed and coordinated the study, RC: performed Vojta method on every subject, MGT: acquisition and collection of data, VP: acquisition and collection of data, FC: participated in the design of the study and performed the statistical analysis, CMS: analysis and interpretation of the data, CR: drafted the manuscript and revised it. All authors read and approved the final manuscript.
